# The Biochemical and Cellular Basis for Nutraceutical Strategies to Attenuate Neurodegeneration in Parkinson’s Disease

**DOI:** 10.3390/ijms12010506

**Published:** 2011-01-17

**Authors:** Elizabeth A. Mazzio, Fran Close, Karam F.A. Soliman

**Affiliations:** Florida A&M University, College of Pharmacy and Pharmaceutical Sciences, Tallahassee, FL 32307, USA; E-Mails: elizabethmazzio@yahoo.com (E.A.M.); fran.close@famu.edu (F.C.)

**Keywords:** Parkinson’s disease, neuroprotective, neuromelanin, nutrition, vitamins

## Abstract

Future therapeutic intervention that could effectively decelerate the rate of degeneration within the substantia nigra pars compacta (SNc) could add years of mobility and reduce morbidity associated with Parkinson’s disease (PD). Neurodegenerative decline associated with PD is distinguished by extensive damage to SNc dopaminergic (DAergic) neurons and decay of the striatal tract. While genetic mutations or environmental toxins can precipitate pathology, progressive degenerative succession involves a gradual decline in DA neurotransmission/synaptic uptake, impaired oxidative glucose consumption, a rise in striatal lactate and chronic inflammation. Nutraceuticals play a fundamental role in energy metabolism and signaling transduction pathways that control neurotransmission and inflammation. However, the use of nutritional supplements to slow the progression of PD has met with considerable challenge and has thus far proven unsuccessful. This review re-examines precipitating factors and insults involved in PD and how nutraceuticals can affect each of these biological targets. Discussed are disease dynamics (Sections 1 and 2) and natural substances, vitamins and minerals that could impact disease processes (Section 3). Topics include nutritional influences on α-synuclein aggregation, ubiquitin proteasome function, mTOR signaling/lysosomal-autophagy, energy failure, faulty catecholamine trafficking, DA oxidation, synthesis of toxic DA-quinones, *o*-semiquinones, benzothiazolines, hyperhomocyseinemia, methylation, inflammation and irreversible oxidation of neuromelanin. In summary, it is clear that future research will be required to consider the multi-faceted nature of this disease and re-examine how and why the use of nutritional multi-vitamin-mineral and plant-based combinations could be used to slow the progression of PD, if possible.

## 1. Introduction

### 1.1. Pathology

The pathology of Parkinson’s disease (PD) involves chronic degeneration of dopaminergic (DAergic) neurons in the substantia nigra pars compacta (SNc). Subsequent decay of the nigrostriatal tract manifests itself clinically by symptomatic rigidity, bradykinesia, postural instability and resting tremor. Prominent pathological manifestations associated with degeneration of SNc DAergic neurons include observations describing mitochondrial abnormalities [1–4], excessive cytosolic dopamine (DA) oxidation, α-synuclein aggregates, autophagolysosome dysfunction, defects in the ubiquitin-proteasome system (UPS), oxidative stress, nitrosative stress, iron released from bound storage and a gradual loss of neuromelanin (NM) [5–7]. These pathological insults are self reinforcing and can advance in cyclical fashion, often intensified by decaying levels of glutathione (GSH), which render greater oxidative damage (via O_2_, H_2_O_2_, OH) [8,9] or lipid/protein nitration (via ONOO^−^) and accumulation of 3-nitrotyrosine, protein carbonyls, 8-hydroxyguanosine, malondialdehyde and hydroxynonenol in degenerating neurons [10–12]. Neurological degeneration can also be aggravated by chronic central nervous system (CNS) inflammation which can involve recruitment of activated microglial cells, release of cytotoxic molecules, free radicals and glutamate, which can provoke neuritic beading, excitotoxic, apoptotic and necrotic degeneration [13]. While gradual loss of DAergic SNc pigmented cells occur as a natural process of aging—early diagnosis of PD is associated with a 30%/60% reduction of DAergic neurons/striatal DA which is attributable to degeneration of striatal axon terminals [14]. Though the etiology circumscribing the selective loss of SNc DAergic neurons in PD is not fully understood, we do know that reportedly ~5–10% of PD patients display mutations in genes such as DJ-1, PTEN-induced kinase 1 (PINK-I), leucine-rich repeat kinase 2 (LRRK2) G2019S, park-1/Synuclein (SNCA), ubiquitin-carboxy-terminal-hydrolase L1, parkin (Del3-5, T240R, Q311X) [15–18], ATP13A2 (Park 9), β-glucocerebrosidase and mitochondrial proteins such as park 13 Omi/Htra2, Complex I [19–22]. The larger majority of PD cases result from a fusion of natural aging and/or environmental exposures to pesticides, a history of depression, viral/bacterial infections, metals, antipsychotic/antidepressant drugs, rural/farm living or lack of habitual cigarette smoking/tobacco use or consumption of caffeine [23–27]. All of these studies provide partial insight as to the precipitating factors involved with PD onset and progression. Moreover, the greatest commonality appears to be either genetic mutations or environmental triggers that lead to direct/indirect accumulation of malfunctional mitochondria, which precede selective DAergic SNc degeneration.

The extent of DAergic SNc losses in human PD can be imaged using positron emission tomography (PET) or single photon emission computerized tomography (SPECT). Radioactive tracers in PD patients have been used to substantiate (**1**) *compromised integrity of pre-synpatic nigrostriatal projections*, *i.e.*, ^[18F]^LDOPA (which monitors DA uptake, metabolism, DOPA decarbarboxylase (DDC), DA storage within intact nerve terminals); (**2**) *faulty DA transporters* (DAT) *i.e.*, CFT, C-RTI-32, FP-CIT ligands, ^[11C]^methylphenidate (MP)/99mTc-TRODAT-1 or (**3**) *abnormal type-2-vesicular monoamine transporter (VMAT2) function* using tracers such as ^[11C]^-dihydrotetrabenazine (DTBZ) which measures cytoplastic DA uptake into synaptic vesicles [28–30]. Chronic SNc DAergic degeneration parallels a reduction of ^[18]^F-DOPA uptake and DAT binding which are foundational events to faulty circuitry in the basal ganglia that ultimately triggers locomotive disability [31].

### 1.2. Treatment

In order to counteract the loss of SNc DAergic neurons, medical treatments are aimed at modulating neurotransmitter (NT) function. Prescription medicines allow for fluid voluntary movement, reduction of tremors and a sustained quality of life. Routine adjunct therapies often combine levodopa/dopa-decarboxylase inhibitors Sinemet^®^ and Madopar^®^ with DA receptor agonists, catechol-*o*-methyltransferase inhibitors, monoamine oxidase (MAO) inhibitors, anti-cholinergics and surgical treatments [32]. While prescription drugs ameliorate the symptoms, they do not necessarily address the central etiology of degeneration and therefore a number of alternative approaches have been considered to slow the progression of this disease.

### 1.3. Previous Studies on Therapeutic Agents to Slow Progression of PD

Innovative strategies to slow the progression have met with partial success in experimental models, and to a less significant extent in clinical trials. Most neuroprotective strategies seem to fall under the general classes of anti-inflammatory, anti-apoptotic, anti-oxidants, enzyme inhibitors, growth factors, alternative medicine or receptor antagonists/agonists. Experimental trials elucidating efficacy of neuroprotective agents are rapidly expanding, and have thus far included superoxide dismutase (SOD)/catalase/peroxidase mimetics [33], anti-apoptotic MAO inhibitors, rasagiline [34–38], (−)-epigallocatechin-3-gallate, iron chelator/antioxidant/anti-inflammatory combinations [39–41], celastrol, nitric oxide synthase (NOS) inhibitors [42,43], COX, c-jun *N*-terminal kinase (JNK) inhibitors [44–47], alpha-tocopherol, coenzyme Q_10_, lipoic acid [48–52], creatine [53,54] melatonin, catalpol from root of *Rehmannia glutinosa Libosch* [55,56], *N*-acetyl-l-cysteine (NAC), thiol antioxidants [57], nerve growth factors [58,59], dehydroepiandrosterone [60], estrogen receptor agonists [61], adenosine A2 receptor antagonists [62–68], S-allylcysteine [66], mGlu2/3 metabotropic [67], acupuncture [68] traditional Chinese medicine Zhen-Wu-Tang [69] angiotensin-converting enzyme inhibitors [70], nicotine, ginseng, ginkgo biloba, caffeine and cannabis [71].

Despite the success using a vast range of therapeutic agents in preliminary experiments, there is a general failure of clinical trials to substantiate therapeutic effects that slow disease progression, in particular for antioxidants. This may be attributable to limitations in the current animal or *in vitro* models that make extrapolation of information for human PD difficult. Further, the pathology is very complex and may not be effectively antagonized with just single therapy antioxidant, ergogenic, anti-inflammatory regimens.

The use of nutritional supplements to slow the progression of PD has also not been fully substantiated by evidenced-based studies. The aim of this review is to re-visit the pathology of PD, and in light of pathological processes further discuss the rationale behind potential use of vitamin/mineral nutraceutical neuroprotective agents. In this review, the details of pathology are presented in Sections 1 and 2, and further discussed relevant to nutrient interactions in Section 3. Discussion includes the role of vitamins and minerals in the established United States recommended daily allowances, as well as macronutrients and plant based constituents that modulate processes with specific relevance to PD. The review is a combination of past literature and proposed theory based on known molecules that affect known biological targets which range from mitochondrial malfunction, inflammation, DA oxidation and defective UPS/lysosomal autophagy processes. Moreover, some of the compounds proposed in this review have not yet been evaluated.

## 2. Review

### 2.1. Energy Failure—Loss of OXPHOS, Rise in Anaerobic Glycolysis & Lactate, ATP Depletion

We first review the most prominent issue underlying the loss of DAergic neurons, which is a fundamental failure in glucose metabolism due to aberration of mitochondrial respiration. It is important to note that mitochondrial malfunction could initially occur due to toxic effects of α-synuclein, endogenous neurotoxins or exogenous environmental factors. However, experimental models often employ use of mitochondrial toxins such as 1-methyl-4-phenylpyridinium (MPP^+^), rotenone or endogenous isoquinolines to selectively target neuropathological damage similar to, *but not identical to* PD degenerative effects mainly in the SNc and the locus coeruelus (LC) [72–74].

Loss of mitochondrial function leads to immediate failure of DA neurotransmission and acceleration of glycolysis to overcome the loss of oxidative phosphorylation (OXPHOS) through substrate level phosphorylation (SLP) [75–77]. The impact of mitochondrial toxins on these energy processes is almost always observed. *In vivo*, administration of 1-methyl-4-phenyl-1,2,3,6- tetrahydropyridine (MPTP) generates an immediate rise in glucose utilization (detected with ^[2–14C]^deoxyglucose), a drop in ATP, a rise in lactate production, reduction in striatal DAT/DA and loss of tyrosine hydroxylase immunoreactivity, effects which are exacerbated by α-synuclein [78–83]. The drop in ATP suggests that energy deficiency is clearly involved with the process of initiating degenerative decline [84]. Moreover, there is ample information to substantiate that a drop in energy corresponds to a rise in glycolysis to drive SLP, an indicator of metabolic stress. The use of proton magnetic resonance spectroscopy (1H-MRS) has been used to confirm sharp spikes in striatal lactate, which occur within 2 h of MPTP injection in C57BL/6 mice [85]. In primates, a long term study utilizing infusion of MPTP over 14 ± 5 months resulted in loss of DA pre-synaptic re-uptake, parallel to a 23-fold increase in striatal lactate production, which was sustained for up to 10 months post final administration of MPTP [86]. While the CNS is most often studied with respect to biochemical effects induced by systemic injection of MPTP, damage in peripheral tissue such as skeletal muscle also involves heightened anaerobic glycolytic function, elevation of lactic acid dehydrogenase and concomitant decrease of mitochondrial Complex IV, with no changes in mitochondrial Complex I [87]. These shifts toward anaerobic metabolism occur in tissues with capability to uptake MPP^+^ where similar patterns observed include loss of ATP, loss of OXPHOS, a rise in glycolysis, heightened production in lactate and neurotoxic effects which can be blocked by providing abundant glucose to growth media in order to sustain ATP production through glycolysis [77,88–93]. The reliance of damaged neurons on greater anaerobic glycolytic function is not exclusive to PD, as head trauma, seizure or ischemia can equally provoke a rise in brain/CSF lactate and loss of ATP parallel to neurological damages [94–98]. In Section 3, we discuss nutrients involved with propelling anaerobic function.

In the human brain, *in vivo* functional imaging strategies to assess glucose metabolism in the brain include PET with a ^[18F]^2-fluoro-2-deoxy-d-glucose (^[18F]^FDG) tracer. This tracer is used to quantify elevated rates of glycolysis/glucose transport relative to surrounding tissue. Some limitations for this method involve the non-specific manner by which ^[18F]^FDG accumulates in the brain. ^[18F]^FDG enters through the glycolytic cycle prior to conversion of pyruvate, therefore its measurement does not differentiate between aerobic (OXPHOS) and anaerobic (SLP) metabolism. Further, uptake is not selective to cell type and therefore false positives (or heightened metabolic activities) are likely to occur in particular for diseases involving active inflammatory tissue, where metabolic rate of glucose is extremely high [99]. This technique however, has been used in sliced striatal tissue to corroborate that regional exposure to MPP^+^ can evokes a sharp rise in cerebral glucose metabolic rate (CMRglc) [93]. FDG PET studies could also be beneficial in terms of evaluating patterns in non-diseased, non-inflammatory models. FDG PET imaging techniques clearly show that the process of aging in monkeys, corresponds to loss in both regional cerebral blood flow/^[15O]^ H_2_O and rCMRglc in many areas of the brain including the cerebellum, hippocampus, striatum, occipital cortex, temporal cortex, and frontal cortex [100]. Age associated hypometabolism in the human brain is also believed to precipitate increased risk for many age associated CNS neurodegenerative diseases [101]. Future research is now considering preventative implementation with nutrients that could assist in minimizing metabolic losses [101].

In brief, the loss of ATP in the SNc is *detrimental* because this single event can initiate a range of downstream collapse on energy requiring systems that can then lead to (1) catecholamine oxidation and formation of DA neurotoxins/free radicals (2) excitotoxic and programmed cell death (3) mitochondrial transition pore opening, matrix swelling, release of mitochondrial proteins into the cytosol, apoptosis [102–107] and microtubular/cell structure collapse [108].

### 2.2. Loss of DA Regulation and Trafficking—VMAT2

One of the first events brought about by reduction of ATP is a loss of energy requiring systems that drive *DA trafficking*. Section 3, also refers to a large number of nutraceuticals that can block these processes. Failure of DA trafficking, not only occurs due to decline in ATP, but can also result from genetic mutations in SNCA (A53T and A30P) [109], mitochondrial insufficiency or oxidative damage by ROS, all which trigger excessive DA release from SNc nerve terminals [110,111]. With regards to energy, a lack of ATP diminishes the capability of intracellular ATPase pumps to sequester DA into synaptic vesicles (where DA is stable due to slightly acidic pH), which is a pivotal factor in initiating a cascade of neurotoxic events [112,113]. Failure of vesicle monoamine transporter 2 (VMAT2) results in immediate leaked DA into the cytosolic compartment (easily subject to oxidative breakdown at neutral pH) where it readily oxidizes to form *neurotoxic DA-quinones,* o*-semiquinones, dopaminergic poisons and related free radicals* [114–116] which can then contribute to eventual decay of the striatal tract [117].

Further, functional loss of VMAT2 can occur due to age-associated losses in VMAT2 *m*RNA expression, which create vulnerability to extensive neurological damage in the presence of mitochondrial toxins such as MPTP *in vivo* or MPP^+^ *in vitro* [118–123]. MPP^+^ can cause further insult due to its ability to bind directly to VMAT2, gain entrance into synaptic vesicles and initiate extrusion of DA back into the cytoplasmic compartment [124,125].

### 2.3. Dopamine Oxidation

Inadequate function or expression of VMAT2 *m*RNA has also been reported in association with PD [126], which could precipitate three main routes by which the oxidation of DA can *become pathological*. These include (1) the enzymatic oxidation of DA via tyrosinase, phospholipase A_2_ (PLA_2_)/prostaglandin H synthase (COX), lipoxygenase and xanthine oxidase to form DA-quinone en route to neuromelanin synthesis (2) non-enzymatic autoxidation of DA by the presence of oxygen, H_2_O_2_, or metals and (3) the enzymatic oxidation of DA by MAO which can lead to H_2_O_2_ production and synthesis of DA-aldehydes. The heavy oxidation of DA (be it non-enzymatic or enzymatic) seems to initiate neurodegenerative pathogenesis, a depletion of glutathione, oxidation of available ascorbate and subsequent oxidative stress in the SNc area [127]. In Section 3, we provide information on nutraceuticals that may be able to antagonize each of the major routes of DA oxidation.

#### 2.3.1. Enzymatic Oxidation of DA, the Neuromelanin Pathway & DA-Quinones

**Figure 3:**
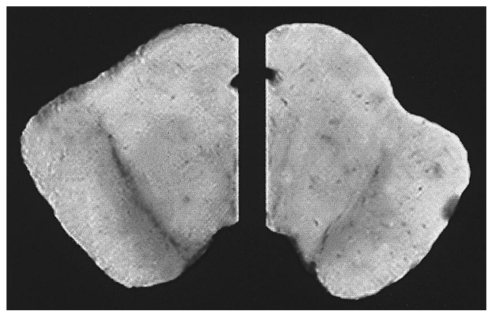
Melanized dopaminergic neurons of the substantia nigra from post mortem human brain. Brain sections taken through the mid- brain of a normal (left) and a Parkinson’s disease patient (right). The Parkinson’s diseased hemisphere on the right shows a loss of the melanized neurons in the substantia nigra in the ventral midbrain [137].

Understanding the role of target enzymes and how they exacerbate DA oxidation could be beneficial in directing future investigation or design of nutraceutical combinations. First, the enzymatic oxidation of DA occurs through heightened activity of tyrosinase, COX, lipoxygenase and xanthine oxidase which converts DA to DA-quinone en route to neuromelanin (NM) [128]. The neuromelanin pathway if intensified can produce deleterious DA-quinone neurotoxic metabolites such as o-semiquinones or benzothiazolines, which are potent inhibitors of mitochondrial pyruvate dehydrogenase (*i.e*., complex I/Krebs cycle) and initiators of α-synuclein fibrillization [129–132]. Oxidized DA can further react with thiols producing DA-cysteine adducts such as 5-S-cysteinyldopamine which mediate metal catalyzed oxidation of proteins, which lead to protein misfolding and aggregation [131]. While gradual accumulation of NM in SNc tissue occurs as a natural process of aging [133], an intense heightened dark melanized pigment (*hyperpigmentation*) appears in the SNc preceding not only neuronal degeneration but also α-synuclein aggregation, inflammation, oxidative stress, apoptosis, Lewy body formation, depletion of GSH, functional loss of DAT and the loss of tyrosine hydroxylase positive neurons [10,128,134,135]. With PD, a biphasic but final loss of NM occurs gradually due to massive oxidation, cell death and release of NM from dying cells [136]. The loss of melanized nigral DAergic neurons is evident in PD brains (Right) when compared to healthy controls (Left) as shown in Figure 3 and is a major part of the pathology [137]. Ultimately, the loss of NM renders failure of its natural protective function, which is to sequester iron, free radicals and toxic quinones [138].

The generation of DA oxidative toxins *also* includes *enzymatic conversion of dopaminochrome to 5,6,-dihydroxyindole* by DT diaphorase, the free radical initiated *conversion of* o*-hydroquinones (protective) to* o*-semiquinones (toxic)* [132] and *transglutaminases* which incorporate sulfur amino acids into DA-cysteine conjugate toxic precursors to neuromelanin [139]. And, recent studies suggest that transglutaminase inhibitors could be useful to prevent cross-linking reactions that lead to neurodegenerative aggregated proteins [140]. Animal models deficient in enzymes capable of catalytically oxidizing DA to DA-quinone (*i.e.*, absent of PLA_2_ COX2), show a resistance to DAergic neurotoxicity after administration of MPTP. This is also corroborated where knockout models for SOD/GSH Px show extensive damage with MPTP [140–144], and protective effects are observed with COX/PLA_2_ inhibitors [145–148].

#### 2.3.2. Non Enzymatic Oxidation of DA, 6-OHDA, Release of Iron & Oxidative Stress

A *second* route for DA oxidation is *non-enzymatically* by reactive oxygen species (ROS) and metals (Fe^2+^, Cu^2+^, and Mn^2+^) [149,150]. The autoxidation of DA can render formation of 6-OHDA (a potent neurotoxin) and O_2_ ^−^. If superoxide reacts with nitric oxide (NO) the formation of ONOO^−^ is evident. Peroxynitrite in turn can cyclically re-oxidize DA, deplete available reduced glutathione/ascorbate (vitamin C), incur a substantial loss of endogenous GSH-peroxidase and destroy the natural ability of GSH to act as an antioxidant [129,151]. While PD patients display depletion of GSH within the SNc [152], the reduction of GSH (*i.e*., γ-glutamylcysteine synthetase inhibitor) in experimental models also renders the SNc vulnerable to the toxic effects of MPTP and 6-OHDA [8]. For this reason, thiol based dietary antioxidants could be considered for clinical trials, as some have reported they prevent MPTP induced toxicity in mice [57,153], attenuate pathological effects of 6-OHDA, ONOO^−^ and block the formation of DA o-semiquinone neurotoxic radicals [154].

Once 6-OHDA is formed, its presence can trigger neurodegeneration through reduction of striatal zinc and metallothione (otherwise antioxidant/metal detoxification agents) and initiate selective release of free iron from ferritin, where pro-oxidant effects predominate [152,155–160]. This could be perilous given the already high concentrations of iron dispersed throughout the substantia nigra, globus pallidus, red nucleus and locus cerulus [161–163]. Heightened free iron deposits are found in the vicinity of neurodegenerative regions, located in microglia, astrocytes and oligodendrocytes in conjunction with a rise in heme oxygenase (HO-1) (an enzyme which yields free Fe^2+^ iron from heme) and disappearance of NM (loss of high affinity binding polymer for Fe^2+^) [164–168]. In PD patients, iron deposition near degenerating neurons [169], could be intensified by hereditary mutations in iron regulatory binding proteins [170,171], iron storage/transport proteins such as ferritin (L/H subunits stabilization: storage/ferroxidase mediated uptake and utilization), caeruloplasmin, iron regulatory protein 2, lactoferrin/melanotransferrin receptors or the divalent metal transporter-1 [172,173]. Further, the accumulation of iron could be heightened by HO-1 which is significantly expressed in SNc dopaminergic neurons, the nigral neuropil, reactive astrocytes and Lewy bodies [174].

To summarize, the dynamics of PD pathogenesis is believed to evolve in part from mitochondrial energy failure, and through a series of events—DA oxidation can perpetuate a cyclical generation of DAergic toxins and precipitate high levels of free iron released throughout the basal ganglia. These events initiate a forward cycle of self-perpetuated DA oxidation, loss of NM and Fe^2+^ mediated damage which can indirectly fuel this loop by additional damage to the 26S proteasome prompting accelerated α-synuclein protein aggregation [175] or production of OH radicals which can oxidize lipids/proteins and DNA [8]. This degenerative cascade could be worsened by genetic mutations in iron transport proteins such as divalent metal transporter 1 (DMT1/natural resistance associated macrophage protein 2/solute carrier family 11, member 2) as noted in the SN of PD patients [176].

In Section 3, we discuss nutraceuticals that could impact each of these processes, in particular focusing on the important role that iron may play in PD pathology [177–179], the removal of which with iron chelators (*i.e*., EGCG, VK-28, clioquincol) is protective in a number of experimental models [40,178,179]. We also discuss the importance of using nutrient based combinations that contain chelators as just one module, since equally destructive forces are contributed by energy compromise, DA oxidation, concentration of free Fe^2+^ and the subsequent down stream metal catalyzed aggregation of insoluble proteins [180].

#### 2.3.3. Enzymatic DA Oxidation by MAO-DA Aldehydes & H_2_O_2_

The third major route for oxidation of DA is through routine deamination by MAO A or B. MAO activity increases with the natural process of aging and can yield toxic products such as hydrogen peroxide (H_2_O_2_), ammonia, aldehydes, reactive oxygen species [180–182], 3,4-dihydroxyphenylacetaldehyde and 3,4-dihydroxyphenylglycolaldehyde. The latter two have been reported to condense with H_2_O_2_ to form *neurotoxic OH radicals* [183,184]. And, in catecholamine neurons, DA can directly react with H_2_O_2_ leading to *formation of 6-OHDA* (neurotoxin) or further condense with acetaldehyde to produce toxic endogenous precursors such as 1,2,3,4-tetrahydroisoquinoline, 1,2,3,4-tetrahydro-β-carboline and R-salsolinol which are then subject to methylation [185–190] by either nicotinamide/salsolinol or phenylethanolamine *N*-methyltransferases forming toxic *N*-methylated pyridines with structure similar to MPTP/MPP^+^ [191,186,187,191].

### 2.4. Excitotoxicity

Mitochondrial energy dysfunction not only leads to collapse of DAergic function, but also instability of neurons to maintain voltage at the plasma membrane. Depolarization can cause over-activation of NMDA receptors throughout the brain, where glycine binds to NR1 and glutamate to the NR2 initiating fast inward Ca^2+^ currents to the cytoplasm. The general theory of excitotoxicity has remained consistent throughout the years and has been described to involve depolarization of the plasma membrane creating excitability in part due to (1) release of Mg^+^ as a voltage dependent *N*-methyl-d-aspartate (NMDA) block at presynaptic receptors (2) greater susceptibility to excitatory postsynaptic inward Ca^2+^ currents in response to glutamate activation on ionotropic NMDA/AMPA/kainate receptors and (3) a loss of inhibitory GABA metabotropic-inward ion currents upon receptor activation.

Mitochondrial toxins such as rotenone can worsen the heightened amplitude of inward ionic currents, effects known to be are reversible by addition of ATP [192]. In terms of circuitry, a deficit of magnesium (Mg) or ATP can lead to failed regulatory control of intracellular Ca^2+^ systems through changes *not only* at the NMDA receptor but also intracellularly through influences on inositol 1,4,5-trisphosphate and ryanodine receptors [193,194]. *In vivo*, studies show that dietary deficiency of Mg lowers NMDA receptor activation threshold and correlates to the overexcitability of glutaminergic neurons [195]. In Section 3, we discuss the importance of dietary Mg, in this and many other processes involved with PD pathology.

In PD, the over-excitability of the NMDA receptor may contribute to neurodegeneration because Ca^2+^ activation of neuronal nNOS can lead to nitrosative stress—a known primary elemental monomer modification leading to toxic mis-folded and aggregated proteins [196,197]. In reciprocal fashion, the accumulation of α-synuclein can stimulate nNOS, caspase-3 and initiate poly(ADP-ribose) polymerase (PARP-1) cleavage, all events which contribute toward neurotoxicity [198]. The toxic effects of α-synuclein on activation of nNOS are corroborated by studies that demonstrate that effects are blocked in the presence of NMDA receptor antagonists such as MK-801 and APV [199].

In addition, mitochondrial toxicity (*i.e*., MPTP) also leads to accumulation of glutamate in the SNc to an extent parallel to degenerative lesion [200]. The rise in glutamate stimulates increase influx of Ca^2+^ calpain activation in the cytosolic compartment, and these toxic effects are reversed by administration of NMDA antagonists, calpain inhibitors *or* antioxidants [201–203]. While the role of the NMDA receptor as it relates to PD is continually debated, it is noteworthy to mention that there is a very delicate balance between preventing over-activation or under-activation of glutaminergic receptors. The function of glutamate in neurotransmission is required for synaptic plasticity. And, as such, some studies also show that NMDA agonists such as D-cycloserine are protective against MPTP induced DAergic degeneration and microglial activation in the brain [204]. So clearly, this topic is very complex.

### 2.5. Inflammation

Both dying neurons and aggregated α-synuclein can trigger local gliosis, microglial activation, T cell infiltration and elevated expression/release of immunological participants [205]. These include major histocompatibility antigens, adhesion molecules, COX-2, IL-1b, IL-2, IL-4, IL-6, TNF-alpha, prostaglandins, glutamate, ROS, iNOS, MPO, NO and O_2_ ^−^ the latter two of which can react forming the neurotoxic molecule ONOO^−^ [205–215]. Many of the inflammatory indicators are found in post-mortem tissue obtained form PD patients, particularly in regions of the SNc, striatum, LC and spinal fluid [216]. Major regulators of this response involve tyrosine kinase, phosphatidylinositiol 3-kinase (PI3K)/Akt, and the mitogen activated protein kinase (MAPK) signaling pathways such as c-Jun NH_2_-terminal Kinase (JNK), extracellular signal-regulated kinases (ERK) ½ p38 MAPK [46,217–222]. MAPK’s are evoked by cytokines or inflammatory stimuli, regulated by protein kinase A/cAMP and ultimately direct gene transcription by phosphorylating nuclear factor-kappa B (NF-κB) [223–226]. PET imaging using PK11195, [1-(2-chlorophenyl)-*N*-methyl-*N*-(1-methylpropyl)-3-isoquinoline carboxamide] show active microglia occurring around neurodegenerative lesions in idiopathic PD patients *vs.* controls [227].

The use of anti-inflammatory agents may attenuate DAergic damage and antagonize global effects through targeting a number of signaling routes such as MAPKs, NF-kappaB activation/nuclear translocation or its association with the CREB-binding protein, IkappaB kinase (IKK), activating protein-1 (AP-1) and/or preventing IkappaB degradation or phosphorylation of c-jun *N*-terminal kinase (JNK) [228–231]. Substances that can inhibit any one of these mechanistic controls should block pro-inflammatory processes and antagonize the formation of iNOS, COX-2, PGE (2) or HO-1, thereby preventing DAergic loss induced by MPTP [232–237]. The co-expression of iNOS and COX-2 could be detrimental because the product formed by NO and O_2_ ^−^ (ONOO^−^) plays a highly relevant role in the pathological processes involved with PD. Removal of one or both of O_2_ ^−^ and NO/ONOO can prevent the deleterious effects of PD model toxins, as reported in transgenic mice deficient in iNOS, nNOS, NADPH oxidase or with overexpression of Mn/SOD conferring resistance to the toxicological effects of MPTP or intrastriatal injection of 6-OHDA [238–243]. Likewise, administration of specific nNOS/iNOS inhibitors or SOD mimetics can protect against the neurotoxicity of MPTP [33,42,244–246]. In the next section, we review nutraceuticals with multi-capabilities on anti-inflammatory signaling processes.

## 3. Nutraceuticals

### 3.1. Energy—Biochemistry and Metabolism

Considering the cascade of degeneration in PD as it involves mutations affecting protein degradation and folding, inadequate energy production in the SNc, DAergic malfunction, degenerative oxidative damage, excitotoxicity and inflammation, the question remains if any nutraceuticals or dietary practices as a lifetime habit can prevent or block one or more of these pathways and as a result slow the progression of PD. The next section provides a review of previous studies and future directives based on mechanisms as discussed above.

#### 3.1.1. Pyruvic Acid

To overcome the *loss of ATP*, due to single or multiple hits directed at the mitochondria within neurons of the SNc, the first question to arise is to elucidate if it is possible to enhance anaerobic capability of the human brain, when physiological control over glucose concentrations are highly regulated. In order to optimize anaerobic glycolysis within the brain, dietary compounds must pass through the blood brain barrier (BBB) and likely work to promote key glycolytic regulators of substrate level phosphorylation including phosphoglycerate kinase, pyruvate kinase or lactic acid dehydrogenase (LDH), which would propel production of ATP [74,247,248]. Nutrient offsets associated with these enzymes are clearly altered during CNS neurological injury as evidenced by significant elevation in the ratios of lactate/pyruvate, NAD^+^/NADH and NADP^+^/NADPH [249]. These nutrient offsets suggest “metabolic stress” and accelerated use of nicotinamide reducing equivalents to drive survival processes to produce ATP through anaerobic shifts, in particular ischemia. The anaerobic process is fueled by the metabolite pyruvate (PY), which is a substrate for the LDH enzyme. Our investigations of the molecule PY, have indicated it to be the most powerful antioxidant of the glycolytic metabolites, also having capability of protect neuroblastoma against MPP^+^/6-OHDA and H_2_O_2_ toxicities *in vitro* [247–250]. It is possible that oral administration of PY could be capable of entering the brain, serving as a substrate for glycolysis, the Krebs cycle and the GABA shunt [251] and for this reason its use has been effective in preventing neurological damage associated with ischemic stroke [252]. Recently, other attributes of PY include an ability to block NMDA excitotoxicity in hippocampal neurons [253]. Although future research will be required to corroborate this, it would seem logical to combine oral administration of pyruvate, w/Mg (a required cofactor for pyruvate kinase) and niacin (precursor of reducing equivalents) which could help to restore nutrient offsets that occur due to accelerated anaerobic glycolysis in the CNS, when oxygen or mitochondrial function is insufficient.

#### 3.1.2. Niacin

Furthermore, a *deficiency of niacin* is known to increase the risk for DAergic neurons to degenerate [191]. Likewise, the toxicity of MPTP is associated with a depletion of niacin, likely due to high demand for NAD^+^ in several biochemical processes including glycolysis and apoptotic over activation of PARP-1 [254–256]. The administration of niacin has shown protective against MPTP induced SNc cell loss and striatal DA depletion *in vivo*, effects which are possibly due to preventing drop in ATP by fueling glycolysis and preventing PARP-I NAD^+^ depletion [257–259]. Further upstream, PARP-1 is under regulatory control of tumor suppressor protein p53, a transcription factor that controls programmed cell death and cell cycle arrest. For this reason, it is not surprising that administration of either *niacin*, *PARP-1/p53 inhibitors* or PARP-1 knockout mice all show a resistance to MPTP mediated DAergic toxicity [256,257,260–262]. Therefore, the use of niacin as a therapeutic agent could be explored further, given its vast biochemical benefits including additional contribution to the pentose phosphate pathway, which regulates endogenous removal of H_2_O_2_ (a major contributing factor to PD pathology) through the GSH-Px system [191]. And, in our previous work, we have also found NADH to be a powerful antioxidant, alone capable of protecting against peroxide induced toxicity in neuroblastoma [247].

With regard to niacin, it is important to make note that a bit of controversy surrounds its use particularly as it relates to PD. Concern has been expressed that its administration could lead to synthesis of endogenous *N*-methylated nicotinamide, a compound with structural similarity to MPP^+^ [263]. Nicotinamide *N*-methyltransferase (NNMT) is the enzyme that can readily convert pyridines to toxic substances very similar to the PD toxic metabolite MPP^+^ [191]. While future research will be required to investigate these concerns, it may be possible to combine administration of niacin with natural compounds known to down regulate NNMT such as plant derived isoquinoline alkaloids, *caffeine* ± precursors (*i.e*., *xanthosine*—green tea or cocoa tea), which reportedly compete for methyl groups otherwise donated by s-adenosyl-l-methionine to drive NNMT enzyme activity [264–266]. The positive effects of caffeine are consistently reported as both administration of caffeine in animal models is therapeutic against MPTP and most importantly human epidemiological studies show that coffee consumption is associated with a decreased risk for developing PD [267,268]. As a side note, magnesium may also be a downregulator of NNMT [269].

#### 3.1.3. Magnesium

Dietary magnesium (Mg) has a vast role in integrated human metabolism and is critically involved with production/utilization of ATP in the human brain. A number of studies suggest that a dietary *deficiency of Mg* is associated with greater loss of DAergic neurons [270]. And. low Mg brain tissue concentrations are evident in human PD patients [271]. With regards to the review on the pathology of PD above, Mg plays *an indispensable* role in proper DA uptake and vesicular storage and transport [272]. Heightened levels of Mg can attenuate effects of Ca^2+^ overload [273,274], augment the function of VMAT2 for sequestration of DA and provide a voltage dependent non-competitive block of the NMDA receptor otherwise responsible for excitability of neurons [199,275,276]. Ample Mg^+^ in the diet could be critical for PD patients, because of its diversity in energy related functions, energy storage processes (phosphocreatine), and its ability to thwart Ca^2+^ mediated neurotoxicity [276,277]. Mg also plays a critical functional role in activation of CuZn-SOD, and could thereby attenuate formation of ONOO^−^, involved with α-synuclein aggregation [278]. As a note, oral administration of Mg could benefit when co-administered with vitamin B6 and vitamin D. both which assist to maximize its adsorption and utilization.

#### 3.1.4. B Vitamins and Regulation of Physiological Homocysteine

Another role for vitamin B_6_ pertaining to the pathology of PD is its conjunction with vitamin B_12_ and folate which regulate homocysteine by aiding its breakdown to methionine and tetrahydrofolate. These effects may attenuate neurotoxicity associated with *hyperhomocysteinemia—*a condition that is not only associated with PD pathology, but also the toxicity of MPTP in experimental models and as a side-effect of L-DOPA [279–281]. High levels of homocysteine could greater the severity of PD because it mediates toxicity by acting on NMDA receptors to precipitate oxidative stress, Ca^2+^ overload and apoptosis [282]. Vitamin B6 could also help to antagonize the hyperhomocysteinemic effects of nicotinamide via enhanced methylation [279]. Folate is another critical nutrient, where deficiencies in human PD patients have been observed in association with greater levels of plasma homocysteine [283,284]. In experimental models, the effects of hyperhomocysteinemia are known to potentiate the neurotoxic effects of MPTP [285]. For this reason, folate, B_12_ and vitamin B_6_ could be combined with a nutraceutical such as betaine and/or serine, which reduce homocysteine levels through aiding in its regulatory conversion to methionine or cysteine, respectively [286,287]. Garlic is another natural compound which can prevent the build up of homocysteine given its ability to stimulate cystathionine β-synthase and inhibit *N*^5^,*N*^10^-methylenehydrofolate reductase [288]. *Further research will be required to explore* the combined use of these particular B-vitamins with some of these nutraceuticals as it relates to homocysteine accumulation and neurotoxicity in human PD.

#### 3.1.5. B Complex Vitamins, Riboflavin and Mitochondrial Disorders

There is also rationale to substantiate use of *B-Complex vitamins* for PD patients, due to the critical role these nutrients play in *glucose metabolism and mitochondrial respiration*. Vitamin B_2_ derivatives such as flavin adenine dinucleotide (FAD)/flavin mononucleotide (FMN) regulate aerobic mitochondrial metabolism by mediating redox reactions through the electron transport chain [74,289]. Interestingly, the use of oral riboflavin supplements in humans can reverse clinical symptoms associated with mitochondrial myopathy/pathologies (involving complex I–II), where reduction of lactate and restored mitochondrial function are associated with clinical improvements [290–294]. And, use of coenzyme Q_10_ (which plays a role in complex I–II function) for treatment of PD has been of considerable interest, although clinical trials have not yet confirmed therapeutic effects [295,258]. The B-Complex vitamins such as thiamin (vitamin B_1_), lipoic acid, biotin, vitamin B_6_, B_12_ folate, and pantothenate work together symbiotically to drive pyruvate dehydrogenase complex, gluconeogenesis and blood, glucose, oxygen delivery to the brain. The B-complex vitamins each play a unique role *of equal importance* but *work collectively* to optimize mitochondrial function, in particular when challenged with toxins such as rotenone [296]. Clinical trials for *multi-vitamin supplements* therefore could be considered.

#### 3.1.6. Creatine, Chromium

Nutraceuticals that optimize ATP storage reserves may further strengthen the capacity of energy requiring systems. Known disturbances in choline/creatine have been observed in PD patients [297], and creatine supplements have been shown to protect against MPP^+^/MPTP, 6-OHDA and glucose deprivation [53,54,298]. However, preliminary studies in our lab have failed to show protective effects by creatine against MPTP induced DA degeneration in the mouse model, a topic under current investigation (unpublished). While creatine could be beneficial in augmenting ATP storage, chromium salts would be equally important in maintaining physiological glucose, glucose tolerance, insulin sensitivity [299] and glycemic functions [300]. Adequate chromium in the diet seems fitting given its role in optimizing systemic glucose metabolism, despite a lack of evidence to suggest chromium aberrations in cerebral spinal fluid of PD patients [301].

### 3.2. Plant Polyphenols—Attenuation of DA Oxidation

The use of vitamins to support energy function could further be combined with plant derived *polyphenolic compounds* (PDPC) that specifically target downstream toxic effects as a direct result to the loss of ATP. These include collapse of DA trafficking, DA oxidation, generation of ROS, fenton reactions, DAergic neurotoxins, loss of NM and CNS glial inflammation. A number of food-based molecules have previously been reported in the literature as being effective in antagonizing specific events within these processes.

#### 3.2.1. Tyrosinase Inhibitors

As stated previously, the initial oxidation of DA to DA-quinone, or from DA quinone to its toxic metabolites are believed to contribute toward DAergic degeneration. Although future research will be required to substantiate this, these processes could be blocked by nutraceuticals such as polyphenolic inhibitors of *tyrosinase*, *COX*, *lipoxygenase*, *PLA_2_*, *xanthine oxidase* or antioxidants/metal chelators.

The first to review is tyrosinase/polyphenol oxidase (PPO) which is a copper requiring metalloenzyme that catalyzes formation of *o*-quinones. A heightened enzyme activity of tyrosinase could be associated with elevated risk for PD [302] and skin hyperpigmentation disorders [303] both which involve heightened oxidation of L-DOPA to form dopachrome [304,305]. These same processes are often researched in the field of food chemistry, due to food browning reactions occurring through PPO enzymes in vegetables such as potato or mushroom. Creatively, it has been proposed that such as model could serve practical for the investigation or screening of nutraceuticals against DA oxidation processes as it relates to PD [306]. Future research could be done to consider analysis of established nutraceuticals known to inhibit tyrosinase, some of which include the following:

#### 3.2.2. COX Inhibitors

Natural inhibitors of COX could also block the initial step of enzymatic DA oxidation to DA quinone through PGH_2_ synthase. A review of the literature shows a number of promising plant derived polyphenolic compounds (PDPCs) as effective COX inhibitors such as:

#### 3.2.3. Lipoxygenase Inhibitors

PDPC’s that may be able to block the initial step of enzymatic DA oxidation to DA-quinone through inhibition of lipoxygenase (5-LOX, 12-LOX) and include the following:

#### 3.2.4. Phospholipase A_2_ Inhibitors

While PLA_2_ inhibitors attenuate DA oxidation reactions, they may serve dual function in PD pathology because they also block formation of arachidonic acid as a substrate for prostaglandins. PLA_2_ inhibitors could be combined with administration of omega-3 fatty acids (*i.e.*, canola/fish oil), thereby reducing PGE_2_ (a pro-inflammatory prostaglandin specifically associated with PD pathology) [377]. Co-administration of vitamin E may enhance absorption of omega-3 fatty acids and prevent fatty acid oxidation. Future research could consider analysis of plant-derived compounds that are known to inhibit PLA_2_ in experimental models of SNc DAergic damage, some of which are known to include:

#### 3.2.5. Xanthine Oxidase Inhibitors

The initial step of enzymatic DA oxidation to DA quinone could be attenuated by xanthine oxidase inhibitors, some of which are known to include the following:

#### 3.2.6. Xanthine Oxidase and Superoxide Scavengers

Combined xanthine oxidase/superoxide scavengers may reduce oxidative stress, prevent formation of ONOO and attenuate the degenerative process, some of which are known to include:

### 3.3. Histidine, Quercetin and Zinc

Other polyphenolic compounds that may block the initial step of enzymatic DA oxidation include substances which down regulate DT diaphorase or mono-oxygenases such as EGCG [432], flavones [433] baicalin, oroxylin-A glucoronides [434], quercetin [435] or histidine [436]. While we mention the protective properties of EGCG and quercetin on PD related processes throughout this review, noted effects of histidine may also include its ability to augment the uptake and transport of zinc into the brain, where zinc can counteract the pro-oxidant effects of iron [437], ischemia-reperfusion [438,439] or mitochondrial toxins such as MPP^+^ [440]. See Section 3.8.

### 3.4. N Acetyl Cysteine

Thiol based compounds are believed to help slow non-enzymatic autoxidation of DA in the presence of ROS and metals (Fe^2+^, Cu^2+^, and Mn^2+^) [149,150]. Autoxidation of DA to 6-OHDA (a potent neurotoxin) and O_2_ ^−^ can be lethal in the presence of NO, forming ONOO^−^. Peroxynitrite can then re-oxidize DA and deplete available reduced glutathione and ascorbate [129,151]. Possible dietary counter intervention could include thiol antioxidants such as NAC which in experimental models blocks the autoxidation of DA, prevents MPTP induced toxicity in mice [57,153] attenuates pathological effects of 6-OHDA, ONOO^−^ and blocks the formation of DA *o*-semiquinone neurotoxic radicals [154].

### 3.5. Hydrogen Peroxide Scavengers

The third route of DA oxidation is through deamination by MAO A or B which yields H_2_O_2_, ammonia [180–182], 3,4-dihydroxyphenylacetaldehyde and 3,4-dihydroxyphenylglycolaldehyde. The latter two condense with H_2_O_2_ to form OH radicals [183,184] and DA reacts with H_2_O_2_ leading to form 6-OHDA or condenses with acetaldehyde to produce toxic precursors subject to methylation [185–190]. Due to the importance of MAO activity and the initial condensation reaction between catecholamines and aldehydes that create precursors subject to methylation, future research could investigate therapeutic food based compounds that work as (1) MAO inhibitors (2) compounds that potentiate aldehyde dehydrogenase such as GSH, NAD^+^ (3) down regulate nicotinate/phenylethanolamine *N*-methyltransferases such as caffeine or 4) scavenge H_2_O_2_.

Removing hydrogen peroxide generated by MAO or DA autoxidation could be very beneficial in slowing the rate of progression in PD. Hydrogen peroxide, if present in high quantities can oxidize DA to 6-OHDA, which in turn can then react with 6-OHDA to propagate OH radicals, contributing to the formation of α-synuclein-Fe aggregates and insoluble filaments [35,441]. The generation of H_2_O_2_ in DAergic neurons initiates multiple degenerative processes such as improper degradation of oxidized proteins through the ubiquitin proteasome pathway, formation of dopachrome and toxic DA quinones [132,442,443]. The role for peroxide in PD pathogenesis is evidenced by the fact that its removal via potentiation of catalase/SOD prevents injury in MPTP models of injury. Transgenic mice that over express cytosolic CuZn-SO/GSH-Px or applied administration of SOD/catalase mimetics (which both dismutase O_2_ ^−^, and convert subsequent H_2_O_2_ to water) provide protection against MPTP, paraquat and 6-OHDA *in vivo* models of injury [33,243,444–446]. In contrast, reduction in GSH-Px/CuZn SOD (*i.e.*, knockout mice) leaves the SNc area vulnerable to oxidative stress and MPTP injury [143,447]. For these reasons, beneficial nutritional substances could include those that upregulate endogenous glutathione peroxidase and/or catalase, such as NAC, GSH, selenium, vitamin E, NADPH and curcumin [448]. Co-administration of niacin (which provides NADPH to drive GSH-Px) along with substances that augment function of GSH-PX could provide synergy in protecting SNc neurons from oxidative stress [57,153]. Other useful nutritional substances could include those that aid in SOD such as methionine, manganese, copper, zinc and propolis [449] and H_2_O_2_ scavengers which are known to include the following:

### 3.6. Iron Chelators

6-OHDA generated during DA oxidation, reduces metallothione and causes release of free iron from ferritin [152,155,160]. Natural substances that antagonize 6-OHDA toxicity such as NAC, GSH, cysteine, pyruvic acid, [455] and zingerone [456] or are integral constituents of metallothioneine such as serine, lysine and cysteine [155] could be further researched. The accumulation of free iron is deleterious because it is associated with degenerating SNc neurons, surrounding glial cells and found after administration of MPTP/6-OHDA in animals [164–169]. Faulty iron homeostasis in the basal ganglia could lead to a number of oxidative reactions, the acceleration of α-synuclein protein aggregation [175] and formation of OH radicals which can damage neuronal lipid/protein and DNA [8]. It is reported that the use of iron chelators protect against MPTP and 6-OHDA models of PD toxicity [40,178,179]. A number of natural substances are capable of reducing/chelating complex iron including the following:

### 3.7. Heme Oxygenase Inhibitors

The accumulation of iron can also occur due to overactivity of the HO-1 enzyme, which can convert heme to free Fe^2+^, and carbon monoxide, this also being significantly expressed in SNc dopaminergic neurons, the nigral neuropil, surrounding reactive astrocytes and Lewy bodies [174]. Up regulation of HO-1 occurs as a natural response to oxidative stress and correlates to iron deposition in the nigral area with degenerative SNc lesions. For this reason, potentially helpful nutritional substances may include those that can inhibit HO-1 directly such as cysteine, resevatrol, vitamin C, sulfur compounds (*i.e.*, NAC, GSH) [471], apigenin [472], quercetin and kaempferol [473].

### 3.8. Zinc and Selenium

While reactive iron contributes to the degeneration in SNc, the administration of zinc (Zn) and selenium (Se) could strengthen combination nutraceutical strategies [474–476]. Dietary intake of Se, Zn are required for the function/expression of endogenous antioxidant enzymes and ample amounts can attenuate iron-induced, MPTP and 6-OHDA induced DAergic degeneration [150,475,477]. Furthermore, chronic inflammation can bring about a Zn deficiency due to the use of Zn-dependent transcription factors that regulate DNA/nucleic acid synthesis in response to cytokine activation in immunocompetant cells (*i.e.*, hypozincemia) [478,479]. A Zn deficiency can also evoke a shift in the ratio of Cu/Zn rending less than normal function of the CuZn SOD, turning it from an antioxidant to a pro-oxidant enzyme [479]. A requirement for zinc in the body could be justified with PD patients, since Zn mediates (a) downregulation of glutamate release, inhibition of NMDA/mGlu-R receptors, protection against NMDA neurotoxicity (b) renders a positive modulation on GABA release (c) stimulates endogenous antioxidant enzymes and nerve growth factors (d) inhibits nNOS, endonucleases, pro-apoptotic cascades (e) augments synaptic plasticity and (f) is known to prevent age related deterioration of learning and memory [437].

Both zinc and selenium contribute to anti-inflammatory effects through downregulation of MAPK p38, JNK and NF-κB DNA binding/AP-1 c Jun activation, where the therapeutic effects of Se also involve a rise in glutathione peroxidase/reduction of lipid peroxidation, increased glucose uptake, ATP production through glycolysis and an anti-apoptotic effects [474,480].

### 3.9. Anti-Inflammatory Nutraceuticals

The CNS inflammatory response is under the ultimate control of kinases such as tyrosine kinase [217], PI3K/Akt, and mitogen activated protein kinase signaling pathways such as JNK, ERK ½ p38 MAPK [46,218–222]. The topic of inflammatory is far too large for this review and therefore is summarized as follows. Briefly, MAPK’s are evoked by cytokines or inflammatory stimuli, regulated by protein kinase A/cAMP and ultimately control gene transcription by phosphorylating NF-κB which then binds to the promoter region of genes to initiate transcription for a range of pro-inflammatory proteins [223–226]. Anti-inflammatory agents can antagonize global effects through targeting a number of these signaling routes such as MAPKs, NF-κB activation/nuclear translocation or its association with the CREB-binding protein, IkappaB kinase (IKK), activating protein-1 (AP-1) and/or preventing IkappaB degradation or phosphorylation of JNK [228–231]. In brief summary, natural substances that may provide protection include those that can inactivate phosphorylated MAPK’s such as ERK ½ kinase, p38 MAPK, JNK, inhibit IkappaB kinase, IkappaB degradation, NF-κB, AP-1 activation, antagonize COX-2/PGE2/iNOS and reduce expression of TNF-alpha and other pro-inflammatory proteins in immuno-competent cells some of which are listed as follows:

Additionally, phosphodiesterase (PDE) inhibitors, in particular PDE 1 and IV through altering cAMP can downregulate iNOS [511] and protect against MPTP toxicity [512]. Food based compounds known to inhibit PDE include the following:

### 3.10. Toxic Protein Aggregates

In this section we briefly discuss a potential for targeted nutraceutical therapies which would prevent accumulation of α-synuclein, augment the ubiquinone-proteasome system (UPS) or inhibit mammalian target of rapamycin (mTOR) signaling to upregulate autophagy, which may in the long term slow the progression of this disease.

#### 3.10.1. Nutraceuticals—Reduction of aggregated α-SYNUCLEIN (PARK1)

In brief, the kinetics of α-synuclein aggregation involves a number of progressive stages some of which could be altered by nutraceuticals. A higher propensity for α-synuclein aggregation can occur due to missense mutations (A30P, A53T, E46K) in human PD [524]. The general kinetics of aggregation involves three stages: (1) a protein monomer must undergo a modification; (2) modified monomers can then readily interact with each other to form small aggregates and (3) aggregates after reaching a certain size, referred to as a “nucleus”, can undergo irreversible rapid volume expansion called elongation which result in the formation of fibrils, then taking up residence as toxic entities in neurons and Lewy bodies. The initial protein modifications can occur due to phosphorylation of α-Synuclein at Ser 129 (p-Ser 129), nitration at tyrosine residues and *C*-terminal truncation- all of which can lead to nucleation where aggregation becomes probable [525,526]. The initial protein modification stage can also occur due to neurotoxic insults including but not limited to DA oxidative products, NO, ROS and high concentration of metals [527–529]. In turn, α-synuclein can directly initiate increased membrane ion permeability, vesicle leakage of DA and decreased mitochondrial respiration [530,531], which in turn can generate compounds that lead to α-synuclein modification. In essence, α-synuclein can lead to toxicity, and neurotoxicity can lead to α-synuclein aggregation. In this review, we have covered information on nutraceuticals that indirectly attenuate events known to evoke the initial stages of propagative α-synuclein misfolding, such as iNOS, nNOS, DA oxidative products and the enzyme pathways by which DA quinones are produced (Sections 3.1–3.9). In addition, nutraceuticals that inhibit enzymes that otherwise phosphorylate α-synuclein such as polo-like kinases (*i.e.*, thymoquinone–black cumin) [532], casein kinase II (*i.e.*, ellagic acid) [533,534], Gprk2GRK2/5 [535] or proteases such as calpains, calcium-dependent non-lysosomal cysteine proteases may prevent a tendency for α-synuclein to aggregate or result in truncated toxins of α-synuclein. The use of any nutri-therapy which can prevent likelihood of aggregation, should lessen cell burden of accumulated insoluble proteins which otherwise has affinity for lipids, presynaptic vesicles, membranes and can cause considerable damage to organelles including mitochondria [536].

#### 3.10.2. (Parkin) E3 ubiquitin ligase and Proteosomal Dysfunction

Once α-synuclein aggregates are formed, a second vulnerability for continued accumulation would be improper recognition and ubiquitination of specific target proteins for degradation by the proteasome. This can occur in part due to genetic defects in parkin-E3 ubiquitin ligase or its associate SCF complex (Skp1-Cullin-F-box protein complex) [537,538]. While nutritional constituents may not be able to halt faulty processes in ubiquitination, it may be possible to optimize the function of the proteasomal complex with dietary agents.

The proteasomal complex consists of a 20S proteolytic core with two 19S regulatory caps, responsible for recognition, proteolysis, unfolding and transport of proteins into the core lumen for processing. Inhibiting the function of the proteasome with lactacystin, PSI or MG-132 can effectively mimic PD pathology including selective SNc degeneration, α-synuclein positive inclusion like granules and activation of glial cells [539,540]. Nutraceutical substances such as iron chelators can protect against the adverse effects of such proteasomal inhibitors with capability to prevent lactacystin-induced DA neurodegeneration *in vivo* [541]. These protective effects are likely because the proteasome can also be adversely affected or inhibited by DA oxidative metabolites, DA quinones or ROS, effects that are also blocked by antioxidants such as GSH, ascorbic acid, vitamin E, SOD or catalase [542]. In this aspect nutraceuticals could serve useful to protect against further insult to an already vulnerable proteosomal complex, not only due to mutations in parkin, but also due to lack of endogenous proteasome activator PA28 expression in the SNc, concomitant to reduced function of α-subunit of the 20S proteasome in the SNc of sporadic PD patients [543,544].

#### 3.10.3. Nutraceuticals, Autophagy and mTOR signaling

A second degradation route for eliminating α-synuclein aggregates and malfunctional mitochondria is through the process of autophagy. The removal of depolarized damaged mitochondria is mediated through a process called mitochondrial fission which is regulated by membrane constriction through dynamin-related protein (Drp1) mitochondrial fission 1 and GTP hydrolysis [545] in preparation for clearance through autolysosomes. Notable mutations in PINK1 can adversely affect this process, by preventing both Drp1-dependent fragmentation and phosphorylation/relocation of Parkin to mitochondria where it then fails to catalyze mitochondrial ubiquitination, recruitment of ubiquitin-binding autophagic components, HDAC6 and p62, and subsequent mitochondrial clearance [546]. Together, genetic mutations in both PINK1 and Parkin lead not only to failure of mitochondria, but also a lack of mitochondrial quality control for proper degradation of mitochondria that are no longer functional. While nutritional constituents may not be able to reverse protein defects associated with function of Park and PINK1 mutations, dietary factors can largely influence and activate autophagy-lysosomal function.

Autophagy is described as the means by which cells degrade oxidized and damaged membranes, organelles and mis-folded proteins. This process is initiated by formation of a phagophore which expands and engulfs portions of the cytoplasm then forming a autophagosome [547]. The initiation stages of autophagosome formation is under control of signaling by class III phosphoinositide 3-kinase and Atg 6 (Beclin-1), which regulates phosphorylation of microtubule-associated protein 1 light chain 3LC3 [548]. These phosphorylated LC3 marked vesicles are then trafficked along microtubules in a dynein reliant fashion and eventually fuse with lysosomes (autolysosomes), where contents are degraded by acidic lysosomal hydrolases. Lysosomes can also reach out on their own in a process called microautophagy where they directly engulf cytoplasm by invagination or septation. And, once inside the lysosome, cathepsin D becomes the main lysosomal enzyme involved in the degradation of α-synuclein [549].

The process of UPS and the autophagy-lysosomal systems are under direct control of mammalian target of rapamycin (mTOR) signaling. Stimuli that lead to upregulation of mTOR serve to block autophagy-lysosomal function and its contribution toward accumulated oxidized and damaged organelles/proteins. Signals that upregulate mTOR include those registering as high nutrient energy status signals, such as glucose, insulin, a high ratio of ATP/AMP ratio, leucine, oxidative stress [550], arginine [551] and high levels of amino acids [552]. The rise in mTOR and reduction in the autophagy-lysosome pathway can be chemically induced by 3-methyladenine or chloroquine, effects which lead to accumulation of Ser-129-phosphorylated α-synuclein [553]. This is opposite to the effects of rapamycin which through inhibition of mTOR activate clearance of aggregate-prone proteins, including α-synuclein as well as faulty mitochondria and prevent the toxic effects of proteosomal inhibitors on DAergic systems [554]. A number of substances in the diet are known to upregulate autophagy lysosomal function by downregulation of mTOR, some of which include resveratrol, spermidine, curcumin, piperine, caffeine, epigallocatechin gallate, garlic, S-allylcysteine [555–557], anthocyanins [558], selenium [559], eicosapentaenoic acid and lycopene [560]. Also, it is likely that nutraceuticals that could selectively inhibit IMPase, IP3, adenylate cyclase [561] or Akt signaling may downregulate mTOR and induce autophagosomal clearance [562].

## 4. Conclusion

In conclusion, this review provides information on nutritional biochemistry as it relates to pathological processes inherent to PD. PD pathology involves both regional and systemic nutrient offsets that are largely related to heightened anaerobic glycolysis, homocysteine metabolism, faulty aerobic energy metabolism, metabolic stress, iron deposition and catecholamine mediated oxidative stress. These offsets could be aggravated by blood -tissue nutrient deficiencies as commonly reported in human PD patients or the process of aging itself, both which could exacerbate protein mis-folding/aggregation, disruption of proteasomal processes or losses in DAergic neurotransmission. Future research will be needed to investigate a strategic means to employ combined nutraceuticals that work effectively and collectively to alter metabolism or pathological processes in such a way as to slow the progression of PD in humans. Any therapeutic strategy that can effectively do so, will afford extended quality of life to human PD patients.

## Figures and Tables

**Figure 1:**
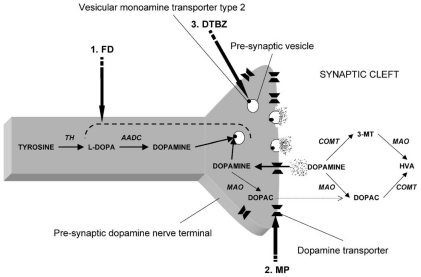
PET Imaging Tools Used in PD. Schematic representation of dopamine synthesis and metabolism, including sites of action of pre-synaptic dopaminergic PET ligands. (**1**) FD reflects uptake of l-dopa, the AADC activity, and the storage of dopamine in pre-synaptic vesicles; (**2**) MP binds to the dopamine transporter, which is specific for the gradient-determined re-uptake of dopamine; and (**3**) DTBZ binds to vesicular monoamine transporter type 2, which is responsible for the uptake of monoamines into pre-synaptic vesicles. In the striatum, more than 95% of the monoaminergic nerve terminals are dopaminergic. (AADC: aromatic amino acid decarboxylase; COMT: catechol-*O*-methyltransferase; DOPAC: 3,4-dihydroxyphenylacetic acid; DTBZ: ^[11C]^-dihydrotetrabenazine; FD: 6-^[18F]^-fluoro-ldopa; HVA: homovanillic acid; l-DOPA: l-3,4-dihydroxyphenylalanine; MAO: monoamine oxidase; MP: ^[11C]^-d-threomethylphenidate; 3-MT: 3- methoxytyramine; TH: tyrosine hydroxylase) [30].

**Figure 2:**
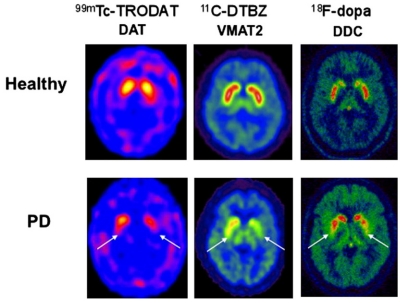
Imaging dopamine terminal function in healthy controls and early Parkinson’s disease (Modified from [28]).

**Figure 4:**
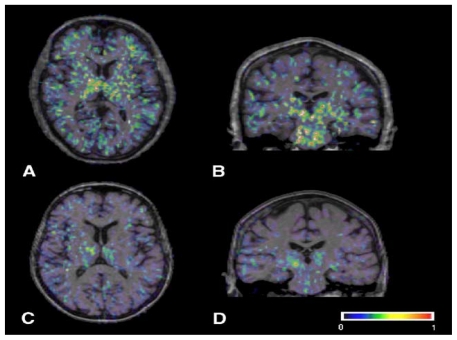
In the PD patient (**A** and **B**), binding is increased in the basal ganglia, pons and frontal regions, while the healthy control person (**C** and **D**) only shows constitutive [11C] (R)-PK11195 binding in the thalamus and pons. The color bar denotes binding potential values from 0 to 1 [227].

**Table 1:** Tyrosinase Inhibitors.

Tyrosinase Inhibitors	Reference
Tetrahydroxychalcones, Butein	[307,308]
Prenylated flavonoids, Sanggenon D	[309]
Sophoraflavanone G, Kuraridin, Kurarinone, Norkurarinol	[310,311]
Cinnamic acid, Aloin, Sophorcarpidine	[312,313]
Glabrene/Licorice, licuraside, isoliquiritin and licochalcone	[314,315]
Quercetin, Galangin, Morin, Fisetin, Luteolin, Apigenin,	[316]
Esculetin	[317]
Hexylresorcinol, Dodecylresorcinol	[318]
Oxyresveratrol	[319]
Gnetol	[320]
(−)-Epigallocatechin-3-gallate, Hinokitiol (beta-thujaplicin), Kojic acid	[321,322]
Reduced glutathione, cysteine, thiol compounds, ascorbic acid, acetic acid	[323–326]
Dimethylsulfide	[327]
Phytic acid	[328]
Tannic acid	[329]
Nobiletin	[330]
Kaempferol	[331,332]
Extract of hibiscus, carex pumila, and garcinia subelliptica	[333]
Wine phenolics	[334]
Green tea	[335]
Procyanidins, Grape seed extract	[336,337]
Gallic acid derivatives	[338]
Safflower	[339]
Aisic acid	[340]
Olive oil constituents	[341]

**Table 2:** Cycloxygenase I/II Inhibitors.

Cycloxygenase I/II Inhibitors	Reference
Quercetin, Kampferol, Chrysin and Galangin	[342,343]
Anthocyanins, Delphinidin, Cyanidin, Malvidin	[344,345]
Galangin, Morin, Apigenin, Rutin, Catechin, EGCG, Quercetin, Chrysin, Flavones, Luteolin, Tectorigenin, Bilobetin, Nobiletin, Fisetin, Naringenin, Quercetin, Lonchocarpol, Tomentosanol and Wogonin	[346–349]
Quercetin, Quercetin 3-glucuronide, Quercetin 3′-sulfate 3′-methylquercetin 3-glucuronide	[350,351]
Ursolic acid, Eugenol, Pyrogallol and Cinnamaldehyde	[352]
Ipriflavone, Resveratrol, MSV-60, Amentoflavone, Ruscus extract	[353,354]
Notoginseng Prenylated flavonoids, Morusin, Kuwanon C, Sanggenon, Kazinol, Kuraridin, Kurarinone, Sophoraflavanone G	[355]
Butein and 7,3′,4′-trihydroxy flavone	[356]
Coumarins, Bergapten	[357]
Amentoflavone	[358]
Oroxylin A	[359]
Caffeic acid Phenethyl Ester and Propolis	[360]

**Table 3:** Lipoxygenase Inhibitors.

Lipoxygenase Inhibitors	Reference
Luteolin, Baicalein, Fisetin, Quercetin, Eugenol, Curcumin, Cinnamaldehyde, Piperine, Capsaicin, Allyl sulfide, Oroxylin A, Wogonin	[361–364]
Morin, Galangin, Kaempherol, Taxifolin, EGCG, Esculetin, Propyl gallate	[365–367]
Coumarin, 7-hydroxy-derivative, Fraxetin, Daphnetin, Coumarin derivatives	[368]
Amentoflavone	[369]
Kuraridin, Sophoroflavonone G, Kenusanone A, Psoralidin	[370]
3,5,6,7,3′,4′-hexamethoxyflavone, Sinensetin, Nobiletin, Tangeretin, Rhamnetin Tetramethylscutellarein, 6,7,8,3′,4′-heptamethoxyflavone, Hesperidin, Ferulic acid	[371]
Sophoraflavanone G, Quercetin, Kenusanone A	[372]
Circiliol, Hypolatein, Sideritloflavone	[373]
Silymarin	[374]
Bean (*Phaseolus vulgaris* L.) hulls	[375]
Cirsiliol, Hypolaetin, Hypolaetin-8-*O*-beta-d-glucoside, Gossypetin, Gossypin, Hibifolin, Leucocyanidol	[376,377]
Oroxylin A, Baicalein, Wogonin	[378]
Procyanidins	[379]
Quercetin glycosides	[380]
Entaureidin and 5,3′-dihydroxy-4′-methoxy-7-carbomethoxyflavonol	[381]

**Table 4:** Phospholipase A_2_ Inhibitors.

Phospholipase A_2_ Inhibitors	Reference
Quercetin. Kaempferol, Myrecetin, Kaempferole-3-galactoside, Scutellarein, Ochnaflavone, Amentoflavone, Ginkgetin, Isoginkgetin, Morelloflavone, Bilobetin, Prenylated flavonoids	[342]
Ginkolide	[378]
Amentoflavone, Ginkgetin	[379]
Fish oil, Evening primrose oil	[380,381]
2′,4′,7-trimethoxyflavone	[382]
Nobiletin	[383]
Rosmarinic acid	[384]
Omega-3 fatty acids	[385]

**Table 5:** Xanthine Oxidase Inhibitors.

Xanthine Oxidase Inhibitors	Reference
Skull Cap (Scutellaria baicalensis (SbE)), Grape seed proanthocyanidins	[386]
Hesperitin, Theaflavin-3,3′-digallate, Cranberry juice	[387–389]
Chrysin, Phloretin, Luteolin, Kaempferol, Quercetin, Myrecetin, Galagin, Apigenin, Morin, Isorhamnetin, Fisetin, Rutin	[390–395]
EGCG, 4-*t*-butylcatechol, Catechin, Fisetin, Luteolin, Raxifolin	[395,396]
Quercetin glycosides	[397]
Apigenin, Quercetin, Isovitexin	[398]
Hydroxyl or Methyl Chalcones (*i.e.*, 3,3,4,4-tetrahydroxychalcone), Esculetin, 4-methylumbelliferone	[399]
Propolis, Caffeic acid phenetyl ester, Chrysin, Galangin	[400,401]
5,7,4′-Trihydroxy-6-methoxyflavone *p*-coumaric acid derivatives drupanin, 4-acetyl-3,5-diprenylcinnamic acid, *trans*-ferulic acid *O*-hexan-3-onyl-ether	[402]
Baicalein, Wogonin, Baicalin	[403–405]
Pycnogenol, Silymarin, Silybin, Silybin flavones, Purpurogallin	[406,407]
Black Tea	[408]
Procyanidins, Pygnogenol	[409–412]
Anthocyanins, Cyanidin, Cyanidin 3-*O*-beta-d-glucoside	[413]
Myricetin Glycosides	[414]

**Table 6:** Xanthine Oxidase and Superoxide Scavengers.

Xanthine Oxidase and Superoxide Scavengers	Reference
EGCG, EGC, Pyrogallol, Catechin, Luteolin, Myrecetin, Rutin, Apigenin, Quercetin, Hesperitin, Naringenin, Biochanin, Retinol, Daidzein, Genestein, 4-*t*-butylcatechol, Taxifolin, Fisetin, Kaempferol, 5,7,4′-trihydroxy-6-methoxyflavone	[395,402,415–417]
Caffeic acid, Rosmarinic acid, Salvianolic acid, Sage	[418]
Apigenin, Quercetin, Diosmin	[419]
Green tea polyphenolics, Theaflavin, EGCG	[388,389,420,421]
Scutellarin	[422]
Oligomeric proanthocyanidins, EGCG, Delphinidin, Myrecetin, Gallic acid, Caffeic acid, Fisetin, Quercetin, Catechin, Epicatechin	[423]
Galangin/Caffeic acid phenethyl ester, Propolis, Caffeic, Chlorogenic acid, Gallic acid	[401,424,425]
Baicalein, Baicalin, Morin	[404,426,427]
Uric acid	[428]
Chrysoeriol ± glycoside	[429]
Anacardiaceae spice	[430]
Myrecetin, Fisetin, Quercetin	[431]

**Table 7:** Peroxide Scavengers.

Peroxide Scavengers	Reference
Acacetin, Dihydrorobinetin, Fisetin, Isorhamnetin, Robinetin, Myricitrin, Hyperoside	[450]
Resveratrol, Catechin, Gallocatechin	[451,452]
Pygnogenol, Pyrogallol, Gallic acid, Anthocyanidins	[452,453]
Gallic acid, Trolox, Kaempferol	[454]
Vanillic/Caffeic acids	[450]
Baicalein	[448]
Hydroxytyrosol	[442]

**Table 8:** Iron Reducing/Chelating Compounds.

Iron Reducing/Chelating Compounds	Reference
Rutin, Morin, Rosemary, Sage, Oregano	[457]
Phytic acid, Brown rice bran, Tannic acid	[449]
Apigenin, Diosmin, Phloretin, Fisetin, Taxifolin, Naringenin	[458]
Quercetin, Rutin, Myrecetin, Luteolin, Epicatechin Caffeic acid, Catechin, Kaempferol, Naringenin, Baicilein	[459–464]
Theaflavin, Theaflavin Digallate	[455,465,466]
Vitamin E, Zinc	[467]
Gallic Acid	[468]
Silymarin, Silybin	[469]
Rutin	[470]

**Table 9:** MAPK/NF-κB/iNOS/COX-2 (−).

MAPK/NF-κB/iNOS/COX-2 (−)	Reference
Selenium, Zinc	[429,435]
Chrysin, Quercetin, Galangin, Propolis or its derivatives	[481–486]
Apigenin	[487,488]
Luteolin	[487,489,490]
Diosmetin, 3-hydroxyflavone, Pillion,4′,7′-dihydroxyflavone, Ayanin, Luteolin, Tectochrysin, 3′,4′-dihydroxyflavone, Tamarixetin, Genestein, Kaempferol, Izalpinin, Ombuine, Biochanin, Tectorigenin, Daidzein, 7-hydroxyflavone, Rhamnetin, flavone, EGCG, Mearnsetin, Liquiritigenin, Myrecetin	[491]
Hydroxychalcones	[228,492,493]
EGCG/Green tea	[494–496]
Butein	[497]
Anthocyanins	[344,498]
5,6,3′,5′-tetramethoxy 7,4′-hydroxyflavone, Artemisia Absinthium, Wormwood, Blackwalnut	[235]
Scutellarin	[499]
Isovitexin	[500]
Naringin, Hesperitin and Naringenin	[501–503]
Baicalein	[504,505]
Silibinin, Silymarin	[225,506,507]
Amentoflavone	[508]
Licorice	[509]
Wogonin	[510]
Curcumin, Luteolin, Wognonin, Kaempferol, Nobiletin, Bilobetin	[342]

**Table 10:** Phosphodiesterase Inhibitors.

Phosphodiesterase Inhibitors	Reference
Butein	[513]
Cirsimarin	[514]
Grape Skins, Anthocyanin, Malvidin	[515]
Diosmetin, Luteolin, Apigenin, Quercetin, Myrecetin	[516]
(+)-Catechin, Caffeic acid	[517]
Gingko Biloba	[518]
Biochanin A, Tyrphostin, Diadzein	[519]
Theophylline	[520]
Amentoflavone, Bilobetin, Sequoiaflavone, Ginkgetin, Isoginkgetin	[521]
Scutellarein, Phloretin, Naringenin	[522,523]

## Abbreviations

6-OHDA: 6 Hydroxydopamine
AADC: Aromatic amino acid decarboxylase
AMPA: α-amino-3-hydroxyl-5-methyl-4-isoxazole-propionate
AP-1: Activator protein 1
ATP: Adenosine triphosphate
BBB: Blood brain barrier
CBF: Cerebral blood flow
CMRG: Cerebral metabolic rate of glucose
CMRG: Cerebral metabolic rate of glucose
COMT: Catechol-*O*-methyltransferase
COX: Cyclooxygenase
CSF: Cerebral spinal fluid
DA: Dopamine
DAergic: Dopaminergic
DAT: Dopamine transporter
DOPAC: 3,4-dihydroxyphenylacetic acid
DTBZ: ^[11C]^-dihydrotetrabenazine
ERK: Extracellular signal-regulated kinases
FAD: Flavin adenine dinucleotide
FD: ^[18F]^-fluoro-l dopa
FDG: ^[18F]^-Fluoro-deoxyglucose
FMN: Flavin mononucleotide
GABA: Γ-Aminobutyric acid
GSHPx: Glutathione peroxidase
GSH: Reduced Glutathione
H_2_O_2_: Hydrogen Peroxide
HO-1: Heme oxygenase 1
HVA: Homovanillic acid
IKK: I kappaB kinase
IL: Interleukin
INOS: Inducible NOS
mRNA: Messenger ribonucleic acid
mTOR: Mammalian target of rapamycin
NA: Nucleus accumbens,
NAC: N acetyl L cysteine
NAD^+^: Nicotinamide adenine dinucleotide
NADH: Nicotinamide adenine dinucleotide reduced
NADPH: Nicotinamide adenine dinucleotide phosphate reduced
NF-κB: Nuclear factor-kappa B
NM: Neuromelanin
NMDA: *N*-methyl-D-aspartate
NNMT: Nicotinamide *N*-methyl transferase
NOS: Nitric oxide synthase
NT: Neurotransmitter
O_2_^−^: Superoxide
OXPHOS: Oxidative Phosphorylation
PARP-1: Poly [ADP-ribose] polymerase 1
PD: Parkinson’s disease
PDE: Phosphodiesterase
PDPC: Plant derived polyphenolic compounds
PET: Positron emission tomography
PGE2: Prostaglandin E2
PGH2: Prostaglandin H2 synthase
PI3K: Phosphoinositide 3 kinase
PINK-1: PTEN-induced putative kinase 1
PK: Pyruvate kinase
PLA_2_: Phospholipase A_2_
PPO: Polyphenol Oxidase
PY: Pyruvate
ROS: Reactive Oxygen Species
Se: Selenium
SLP: Substrate level phosphorylation
JNK: c-jun *N*-terminal kinase
LC: Locus coeruleus
LDH: Lactate Dehydrogenase
l-DOPA: l-3,4-dihydroxyphenylalanine
LOX: Lipoxygenase
MAO: Monoamine oxidase
MAPK: Mitogen-activated protein kinase
MP: ^11C^-methylphenidate
MPP^+^: 1-methyl-4-phenylpyridinium
MPTP: 1-methyl-4-phenyl-1,2,3,6-tetrahydropyridine
SN: Substantia nigra
SNc: Substantia nigra pars compacta
SNCA: α-synuclein
SOD: Superoxide dismutase
SPECT: Single photon emission computed tomography
TH: Tyrosine hydroxylase
TNF: Tumor necrosis factor
UPS: Ubiquitin-proteasome system
VMAT2: Type-2-vesicular monoamine transporter
Zn: Zinc
